# Cladribine treatment in chronic inflammatory demyelinating polyradiculoneuropathy and multifocal motor neuropathy: two case reports

**DOI:** 10.3389/fimmu.2026.1782666

**Published:** 2026-08-05

**Authors:** Konrad Rejdak, Joanna Bielewicz, Marek Kamiński, Paweł Grieb

**Affiliations:** 1 Department of Neurology, Medical University of Lublin, Lublin, Poland; 2 Department of Experimental Pharmacology, Mossakowski Medical Research Institute, Polish Academy of Sciences, Warsaw, Poland

**Keywords:** case report, CIDP, cladribine, immune-mediated neuropathy, multifocal motor neuropathy

## Abstract

Chronic inflammatory demyelinating polyradiculoneuropathy (CIDP) and multifocal motor neuropathy (MMN) are immune-mediated peripheral neuropathies that may follow a progressive course and be refractory and poorly tolerated to standard immunomodulatory treatments. Therapeutic options for such patients remain limited. We report two patients with immune-mediated neuropathies—one with typical CIDP and one with MMN—who were treated with low-dose intermittent subcutaneous cladribine. Clinical outcomes were assessed using the Medical Research Council (MRC) sum score and the Inflammatory Neuropathy Cause and Treatment (INCAT) disability score. Both patients demonstrated clinically meaningful and sustained improvements in muscle strength and functional disability lasting up to 24 months after treatment completion. Cladribine was well tolerated; transient lymphopenia occurred in one patient without serious adverse events. These cases suggest that cladribine may represent a potential therapeutic option for selected patients with CIDP or MMN. Further studies are warranted to confirm efficacy and safety.

## Introduction

Immune-mediated peripheral neuropathies comprise a heterogeneous group of disorders affecting the peripheral nervous system as a result of aberrant immune responses. Disease courses range from acute to chronic and may be monophasic, relapsing–remitting, or progressive (1). Among these conditions, CIDP and MMN are classified as chronic immune-mediated neuropathies and represent potentially treatable causes of long-term disability (2).

Although relevant first-line therapies are effective in many patients, a significant subset remains refractory, intolerant, or dependent on long-term treatment with incomplete functional recovery due to differences in the pathological background (3).

CIDP is the most common chronic neuropathy, characterized by progressive or relapsing symmetric proximal and distal weakness with sensory involvement and reduced or absent tendon reflexes. The clinical spectrum of CIDP is broad and includes several atypical variants, such as distal acquired demyelinating symmetric neuropathy, multifocal acquired demyelinating sensory and motor neuropathy (Lewis–Sumner syndrome), focal CIDP, and pure motor or sensory forms. The immunopathogenesis of CIDP is complex and incompletely understood. Macrophage-associated demyelination is considered a major mechanism, although both humoral and cellular immune responses contribute (4). Recent identification of autoantibodies directed against paranodal proteins such as neurofascin-155 and contactin-1 has led to the concept of nodo-paranodopathies, highlighting pathogenetic heterogeneity within the CIDP spectrum (3).

MMN is a distinct immune-mediated neuropathy characterized by slowly progressive, asymmetric, predominantly distal limb weakness without objective sensory involvement. The presence of persistent multifocal motor conduction block and IgM anti-GM1 antibodies in a substantial proportion of patients supports an immune-mediated mechanism. Unlike CIDP, MMN typically responds to immunoglobulin therapy, whereas corticosteroids and plasma exchange may be ineffective or harmful (5).

These limitations have prompted interest in alternative immunomodulatory and immune reconstitution approaches. One such candidate is cladribine, a lymphocyte-reducing agent widely used in multiple sclerosis (6, 7), whose potential role in peripheral immune-mediated neuropathies has not been systematically explored.

Clinical experience with cladribine in other autoimmune neurological disorders, including myasthenia gravis (8) and neuromyelitis optica spectrum disorder (9), further supports its potential as a repurposed therapy in immune-mediated diseases of the nervous system.

## Case presentation

### Case 1

**Figure 1:**
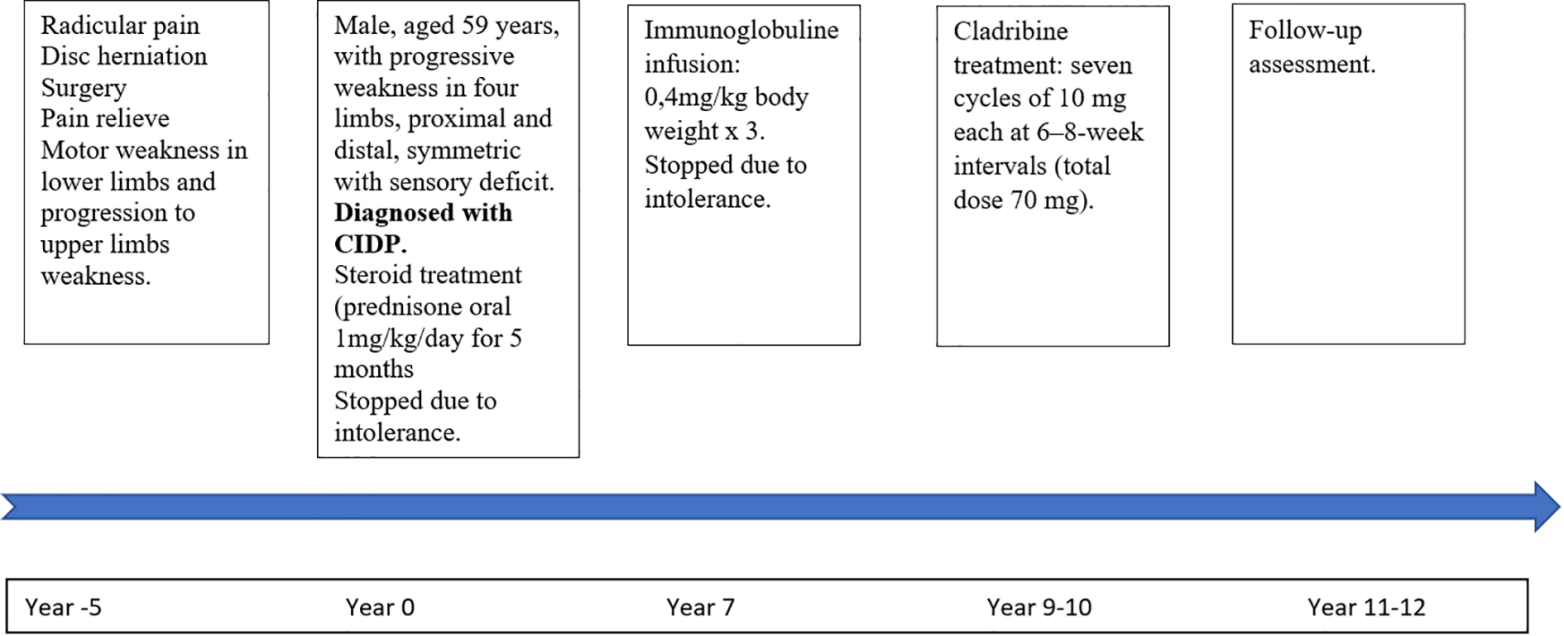
Clinical course and treatment timeline of a patient with chronic inflammatory demyelinating polyneuropathy (CIDP) treated with cladribine.

A 76-year-old man developed progressive muscle weakness and sensory impairment predominantly affecting the lower limbs, with onset at 59 years of age (**Figure 1**). Initial symptoms were attributed to lumbosacral disc disease, and neurosurgical intervention relieved pain without improving motor deficits. Over subsequent years, weakness progressed to involve upper limbs. Electrophysiological studies revealed severe symmetric sensory-motor demyelinating–axonal neuropathy and the diagnosis of CIDP was made. The patient was tested for the presence of paraneoplastic syndrome but the results of chest and abdomen CT scan did not reveal any tumor and a panel of paraneoplastic antibodies in serum was negative. The therapy was initiated and corticosteroid was ineffective (oral prednisone in a dose of 60 mg daily for 5 months) and stopped due to side effects. Intravenous immunoglobulin therapy was tried and discontinued due to severe skin exanthema. The patient was subsequently tested for nodo-paranodopathy antibodies panel including Anti-Neurofascin 155 (NF155), Anti-Neurofascin 186, Anti-Contactin-1 (CNTN1), Anti- Contactin-Associated Protein 1 (CASPR1) and the results were negative.

Given intolerance to standard treatments the patient was not treated for at least 1 year before subcutaneous cladribine was initiated. Before starting treatment, the patient had a moderate neurological deficit in the upper limbs and a significant neurological deficit in the lower limbs: MRC = 41; INCAT = 5 (Arm grade: 2; Leg grade: 3). The patient received a cumulative dose of 70 mg administered as single 10 mg injections every 6–8 weeks (or longer) under the control of blood counts (lymphocyte levels) during in total 2 years of observation. Four months after completion of therapy, substantial improvement in muscle strength and disability was observed: MRC sum score improved to 48 (**Table 1**) and INCAT score to 3 (Arm grade: 1; Leg grade: 2). Transient lymphopenia occurred after the final dose and resolved spontaneously. Clinical improvement was sustained over 24 months of follow-up.

**Table 1:** Clinical characteristics of a patient with chronic inflammatory demyelinating polyneuropathy (CIDP) treated with cladribine.

Before cladribine treatment		
Muscle group tested	Right	Left
Upper arm abductors	5/5	5/5
Elbow flexors	4/5	5/5
Wrist extensors	4/5	5/5
Hip flexors	3/5	3/5
Knee extensors	4/5	3/5
Foot dorsal flexors	0/5	0/5
MRC= 41	20	21
After cladribine treatment		
Upper arm abductors	5/5	5/5
Elbow flexors	5/5	5/5
Wrist extensors	4/5	5/5
Hip flexors	5/5	4/5
Knee extensors	5/5	4/5
Foot dorsal flexors	1/5	0/5
MRC= 48	25	23

The patient received seven subcutaneous cycles of cladribine (10 mg per cycle) at intervals of 6–8 weeks, resulting in a total cumulative dose of 70 mg.

### Case 2

**Figure 2:**
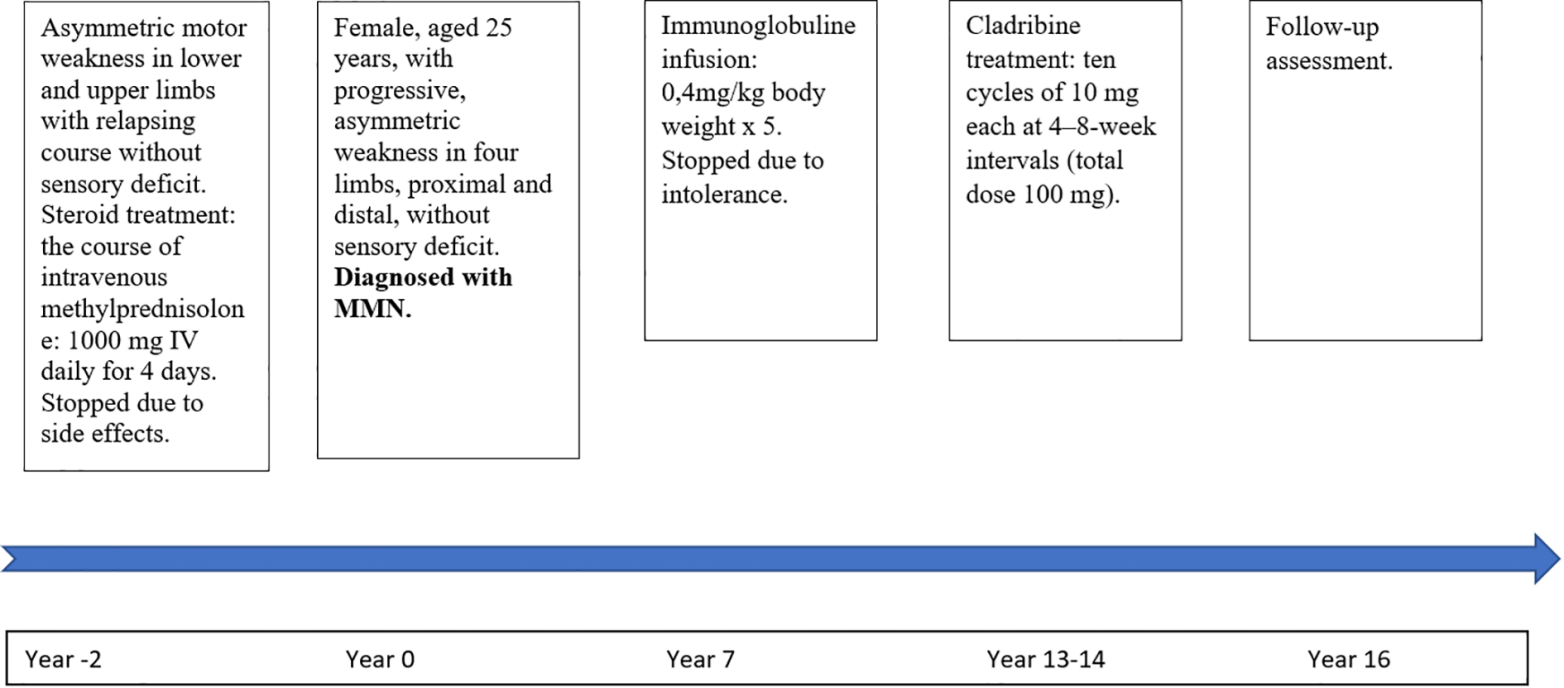
Clinical course and treatment timeline of a patient with multifocal motor neuropathy (MMN) treated with cladribine.

A 39-year-old woman with MMN diagnosed at age 25 presented with progressive asymmetric limb weakness without sensory involvement (**Figure 2**). She had extensive diagnostic work-up which revealed no systemic or hormonal abnormalities and a normal cerebrospinal fluid examination. A nerve conduction study demonstrated motor asymmetric polyneuropathy with conduction block. Initial treatment with corticosteroids (the course of intravenous methylprednisolone: 1000 mg IV daily for 4 days) was ineffective and stopped due to side effects. Intravenous immunoglobulins (0,4mg/kg body weight x 5) were complicated by severe intolerance, including hypertension and dyspnea, precluding continued use. Neurological deficits progressed despite rehabilitation.

Before starting treatment, the patient had a moderate neurological deficit in the left upper limb and a significant neurological deficit in the lower limbs: MRC = 37; INCAT = 7 (Arm grade: 3; Leg grade: 4). Subcutaneous cladribine was initiated, with a cumulative dose of 100 mg administered in cycles of a single subcutaneous injection of 10 mg of cladribine every 4–8 weeks starting under the control of blood counts (lymphocyte levels) during 2 years. After eight treatment cycles (80 mg cumulative dose), marked improvement was evident: MRC 46 (**Table 2**) and INCAT 4 (Arm grade: 2; Leg grade: 2). No lymphopenia or serious adverse events were observed, and improvement was maintained during follow-up of at least 24 months.

**Table 2:** Clinical characteristics of a patient with multifocal motor neuropathy (MMN) treated with cladribine.

Before cladribine treatment		
Muscle group tested	Right	Left
Upper arm abductors	2/5	5/5
Elbow flexors	3/5	5/5
Wrist extensors	3/5	5/5
Hip flexors	2/5	3/5
Knee extensors	3/5	2/5
Foot dorsal flexors	2/5	2/5
MRC= 37	15	22
After cladribine treatment		
Upper arm abductors	3/5	5/5
Elbow flexors	4/5	5/5
Wrist extensors	3/5	5/5
Hip flexors	3/5	5/5
Knee extensors	4/5	4/5
Foot dorsal flexors	3/5	2/5
MRC= 46	20	26

The patient received subcutaneous cladribine in 10 cycles of 10 mg each administered every 4–8 weeks, resulting in a cumulative dose of 100 mg.

## Discussion and conclusions

Both CIDP and MMN involve dysregulated immune responses targeting peripheral nerve structures. While immunoglobulins exert broad immunomodulatory effects, they do not induce durable immune reset and often require chronic administration. Certain lymphocyte-depleting therapies may offer an alternative strategy by interrupting pathogenic immune cascades and enabling immune reconstitution.

Experience with agents such as cyclophosphamide, rituximab, and autologous hematopoietic stem cell transplantation suggests that profound immune modulation can induce sustained remission in selected cases, albeit with significant safety considerations (10). Within this context, cladribine represents an attractive candidate due to its selective and transient effects on lymphocyte populations. Cladribine (2-chloro-2′-deoxyadenosine) is a purine analogue resistant to degradation by adenosine deaminase. Following intracellular phosphorylation, cladribine accumulates preferentially in lymphocytes due to a high cytidine kinase–to–nucleotidase ratio, resulting in selective depletion of T and B cells (11). Its effects extend to both dividing and non-dividing lymphocytes. Cladribine has demonstrated efficacy in multiple sclerosis, where it is thought to act primarily through depletion of memory B cells and long-lasting immune reconstitution. Beyond its cytotoxic effects, cladribine modulates cytokine production and interacts with adenosine A2A receptors, contributing to its immunoregulatory properties (12).

The present cases suggest that cladribine might potentially induce sustained clinical improvement in selected patients with immune-mediated peripheral neuropathies. These observations align with the concept of immune reconstitution therapy, increasingly recognized in central nervous system autoimmunity. High-dose intravenous cladribine has been associated with neurotoxicity (13), highlighting the importance of cautious dosing (14). The low-dose intermittent subcutaneous regimen used here was well tolerated. Previous reports of cladribine use in peripheral neuropathies are scarce and include isolated case of paraproteinemic neuropathy (15). The current dosing was adopted to current needs and characteristics of our patients. Our aim was to continue treatment with small doses given over 1–2 year period and to avoid severe lymphopenia.

The concept of immune reconstitution as a therapeutic strategy in severe immune-mediated neuropathies is supported by the reports of autologous hematopoietic stem cell transplantation (AHSCT) in patients with highly refractory CIDP (16). In a small cohort of five patients with CIDP or Lewis–Sumner syndrome unresponsive to multiple immunomodulatory and immunosuppressive agents, including cladribine and rituximab, AHSCT resulted in marked clinical improvement in four patients, without treatment-related mortality. One patient with longstanding neurogenic atrophy failed to improve, suggesting that irreversible axonal damage may limit therapeutic benefit. Notably, testing for paranodal antibodies was incomplete, raising the possibility that some of these patients represented nodo-paranodopathies distinct from classical CIDP.

To date, no published reports have described cladribine use in MMN (10).

Another interesting treatment indication for this type of intervention could be neurological immune-related PNS adverse events after rare side effects of immune-checkpoint inhibitors (ICIs), which are being used for the treatment of cancer. Indeed, there is an urgent need to search for novel therapeutic agents effective for these debilitating conditions (17).

The above observations further support the notion that profound immune reconstitution might alter disease trajectory in selected patients (18). In this context, cladribine may represent a less invasive immune reduction and subsequent tolerance approach with similar efficacy and good safety profile. However, one must remember that this is a preliminary report and based only on 2 subjects and further studies are needed to test the hypothesis and to provide definite evidence for therapeutic effect.

## Data Availability

The original contributions presented in the study are included in the article/supplementary material. Further inquiries can be directed to the corresponding author.
